# Circulating immune-complexes and complement activation through the classical pathway in myeloperoxidase-ANCA-associated glomerulonephritis

**DOI:** 10.1080/0886022X.2022.2068445

**Published:** 2022-05-02

**Authors:** Tadasu Kojima, Dan Inoue, Takeaki Wajima, Takahiro Uchida, Muneharu Yamada, Isao Ohsawa, Takashi Oda

**Affiliations:** a Department of Nephrology and Blood Purification, Kidney Disease Center, Tokyo Medical University Hachioji Medical Center, Hachioji, Japan; b Department of Microbiology, Faculty of Pharmacy, Meijo University, Nagoya, Japan; c Department of Nephrology, Saiyu Soka Hospital, Soka, Japan

**Keywords:** Anti-neutrophil cytoplasmic antibody (ANCA), myeloperoxidase, circulating immune-complex (CIC), complement, classical pathway

## Abstract

**Background:**

Anti-neutrophil cytoplasmic antibody (ANCA)-associated glomerulonephritis (AAGN) is the fulminant glomerular diseases with poor renal prognosis. Activation of the complement system has recently been reported in the pathogenesis of AAGN, but it remains to be clarified as to which complement pathway is mainly involved.

**Methods:**

20 patients with myeloperoxidase (MPO)-AAGN were retrospectively evaluated. Using serum samples, circulating immune-complexes (CICs) were assessed by the monoclonal rheumatoid factor assay, and C5a and C5b-9 were assessed by ELISA. Complement activation through the classical pathway was further evaluated by the WIESLAB® Complement System Classical Pathway kit. The affinities of ANCAs were evaluated by a competitive inhibition method using ELISA, and were classified into the high, and low-affinity group. Deposition of complement components, such as C3, C5, C4d, C5b-9, factor Bb, mannan-binding lectin serine peptidase (MASP)-1, MASP-2, and mannose/mannan-binding lectin (MBL), in frozen renal sections were analyzed by immunofluorescence staining.

**Results:**

CICs were found to be positive in 65% of the patients. All CIC-positive patients belonged to the high-affinity group. Furthermore, serum C5a and C5b-9 were significantly increased in MPO-AAGN patients, and these levels positively correlated with CIC levels. A significant negative correlation was also found between levels of WIESLAB® classical pathway kit and CICs. By immunofluorescence staining, glomerular deposition of C4d, C5, and C5b-9 were observed in similar distributions in MPO-AAGN patients, whereas the deposition of MASP-1, MASP-2, MBL, and factor Bb were minimal.

**Conclusions:**

These results suggest the involvement of immune-complex induced complement activation through the classical pathway in the pathogenesis of MPO-AAGN.

## Introduction

Anti-neutrophil cytoplasmic antibody (ANCA)-associated glomerulonephritis (AAGN) is a serious kidney disease characterized clinically by rapidly progressive glomerulonephritis (RPGN) and histologically by necrotizing glomerulonephritis with crescents. Although the precise pathogenic mechanism of AAGN has not been fully elucidated, the central role of ANCA has been accepted widely. ANCA is the autoantibody against neutrophil proteins, two major targets of which are proteinase 3 and myeloperoxidase (MPO). Both ANCAs can bind to and activate primed neutrophils expressing target antigens of ANCA on their cell surface, causing respiratory burst with release of neutrophil extracellular traps (NETs), containing DNA fibers, histones, coated with neutrophil derived proteinases such as MPO and neutrophil elastases [1,2]. NETs components are suspected to cause tissue injury and augment autoimmunity, leading to further production of ANCA.

According to the international classification criteria of 2012 revised Chapel Hill Consensus Conference (CHCC2012) [3], AAGN is classified as pauci-immune necrotizing inflammation of the small blood vessels, which means little or no deposition of immunoglobulins or complement components, and hence the role of complements in AAGN has scarcely been reported for a long time. However, several lines of evidence haverecently demonstrated the crucial roles of complement activation in the pathogenesis of AAGN. Several previous studies have indicated that complement activation through an alternative pathway plays an important role in the development of AAGN [4–7]; however, few reports have addressed the roles of the classical complement pathway in AAGN [8]. Considering the recent outstanding advances in the field of complement-regulating therapy, elucidation of the precise complement activation pathway involved in the disease process of AAGN is crucial, because this will clarify therapeutic options for this disease. Indeed, various medical agents that control specific steps of the complement activation pathway, such as pegcetacopan (C3 [9]), eculizumab (C5 [10]), avacopan (C5aR [11]), iptacopan (factor B [12]), danicopan (factor D [13]), etc., are being developed, and are clinically used for some pathogenic conditions. Therefore, the aim of this study was to analyze the existence of circulating immune-complexes (CICs) and the detailed status of complement system activation in patients with AAGN.

## Materials and methods

### Patients and ethics approval

All patients who were newly diagnosed as having MPA based on the CHCC2012 criteria [3] between March 2011 and November 2019 at Tokyo Medical University Hachioji Medical Center were retrospectively reviewed. All patients had MPO-ANCA and showed clinical evidence (glomerular hematuria with proteinuria of more than 0.5 g/gCr), as well as histological evidence of renal involvement (glomerular crescent formation). Patients with other underlying diseases that cause nephritis, such as IgA nephropathy, systemic lupus nephritis, and drug-induced vasculitis were excluded from this study.

All studies were performed in accordance with the principles of the Declaration of Helsinki and with approval from the Research Ethics Committee of Tokyo Medical University (Approval number: T2020-0302). Written informed consent from all the patients included in this study was obtained for the use of their routine clinical test data as well as residual biological samples (serum and renal biopsy tissues) for research.

Serum samples of 10 healthy volunteers from whom written informed consent was obtained were also used as normal controls.

### Data collection

Routine laboratory data at diagnosis, including urinary protein, urinary RBC, serum creatinine, C3, and C4 levels were collected from the patients’ electronic medical charts of our hospital.

### MPO-ANCA titers and their affinity in serum

As the method of measurement and reference ranges of MPO-ANCA of our hospital have changed over time, the titers of MPO-ANCA in stored serum samples of all patients were reassessed using the MPO-ANCA ELISA kit (Phadia GmbH, Freiburg, Germany), generally following the manufacturer’s instructions. At the same time, the affinity of ANCA for MPO was measured by a competitive inhibition method using ELISA, as previously described [14,15], with some modifications. Briefly, the MPO-coated wells of the plate in the MPO-ANCA ELISA kit (Phadia GmbH) were blocked with the blocking buffer Starting Block T20 (Thermo Fisher Scientific, Tokyo, Japan). Then, serially diluted MPO (0–40 μg/mL; 25 μL/well) and diluted serum samples (dilution range from 100× to 2,000×; 25 μL/well) were simultaneously added to the wells and incubated for 1 h at 37 °C. The bound IgG was then detected by incubating with alkaline phosphatase-conjugated anti-human IgG (Sigma-Aldrich/Merck, Tokyo, Japan) for 1 h at 37 °C, followed by color development with substrate (Thermo Fisher Scientific) for 1 h at 37 °C, and measuring of the OD at 405 nm using a plate reader. The competitive inhibition rate was calculated by the following formula.

Inhibition rate (%) at X MPO concentration (μg/mL) = [(OD value without inhibition − OD value with X inhibition)/OD value without inhibition] × 100

An approximation curve was drawn based on the assay results, and then the liquid-phase MPO concentration causing 50% competitive inhibition (IC50) of MPO-ANCA binding to the solid-phase MPO was calculated from the curve. The IC50 results were finally classified into two groups, i.e., the MPO-ANCA high-affinity group and the low-affinity group.

### Circulating immune-complexes (CICs)

Levels of CICs in the stored serum samples of each patient were measured by the monoclonal rheumatoid factor (mRF) assay (CIC-mRF) using an ELISA kit (Nissui Pharmaceutical Co., Ltd., Tokyo, Japan), and by the C1q binding assay (CIC-C1q), also using an ELISA kit (Fujirebio Diagnostics, Inc., Tokyo, Japan), both of which were performed by a clinical laboratory testing company (SRL, Inc., Tokyo, Japan). The reference ranges for these assays were less than 4.2 μg/mL and 3.0 μg/mL or less, respectively.

### Complement components in serum

Levels of serum C5a and C5b-9 were evaluated using an ELISA kit (Quidel Corporation, OH, USA), generally following the manufacturer’s instructions. Because reference ranges for these assays were not provided by the manufacturer of the kits, the serum of 10 healthy volunteers were simultaneously assessed as controls. At the same time, complement activation through the classical pathway in serum was evaluated by WIESLAB® Complement System Classical Pathway (Svar Life Sience Co., Malmö, Sweden), generally following the manufacturer’s instructions.

### Routine histological analyses

Results of routine histological analyses of renal biopsy tissues, such as light microscopic findings of formalin fixed paraffin embedded tissue sections, immunofluorescence staining for IgG, IgA, IgM, C1q, and C3c on fresh frozen tissue sections, and electron microscopic findings were collected from the patients’ medical charts in our hospital. Regarding the light microscopic findings, in addition to the usual evaluation items such as glomerular global sclerosis rate and crescent formation rate, we also evaluated the interstitial fibrosis and tubular atrophy (IFTA) score as the chronic histological change using the previously reported grading scale of 1–4 [16].

### Additional histological analyses of complement components on fresh frozen tissue sections

Sections of fresh frozen renal biopsy tissues from all patients were analyzed for the deposition of complement components in renal tissues. First, direct double staining for C3 and C5 were performed. For this, a rabbit polyclonal antibody against human C3 (pan-specific for C3; ICN, Costa Mesa, CA, USA) was conjugated with Alexa Fluor 594, and humanized mouse monoclonal anti-human C5 antibody (eculizumab; Alexion Pharmaceuticals, Tokyo, Japan) was conjugated with Alexa Fluor 488 using a protein labeling kit (Molecular Probes, Inc., Eugene, OR, USA) according to the manufacturer’s instructions. Then, both labeled antibodies were simultaneously applied and incubated on the sections, washed with PBS and mounted. Indirect double immunofluorescence staining was also performed for C4d and C5b-9 using the polyclonal rabbit anti-C4d antibody (American Research Products, Inc., MA, USA) and monoclonal mouse anti-C5b-9 antibody (Abcam, Cambridge, UK) as the primary antibodies, together with the appropriate secondary antibodies (Alexa Fluor 594-conjugated donkey anti-rabbit IgG antibody for C4d, and Alexa Fluor 488-conjugated donkey anti-mouse IgG antibody for C5b-9; Molecular Probes). Indirect immunofluorescence staining for factor Bb, mannan-binding lectin serine peptidase (MASP)-1, MASP-2, and mannose/mannan-binding lectin (MBL) using the mouse anti-factor Bb neoantigen monoclonal antibody (Quidel Corporation), rabbit anti-MASP-1 and MASP-2 polyclonal antibodies (Sigma-Aldrich/Merck, Darmstadt, Germany), and mouse anti-MBL monoclonal antibody (Abcam) as the primary antibodies, respectively, and Alexa Fluor 488-conjugated donkey anti-mouse IgG antibody (Molecular Probes) or Alexa Fluor 488-conjugated donkey anti-rabbit IgG antibody (Molecular Probes) as the secondary antibodies. All of the glomerular immunofluorescence staining results were semi-quantitatively classified into five grades, as −, ±, +, ++, and +++, and intensities higher than + were defined as positive.

### Statistical analysis

All statistical analyses were performed using SPSS software (version 27 for Mackintosh; IBM Corp., Armonk, NY, USA). Data were expressed as the median with interquartile range. The Mann–Whitney *U*-test, Fisher's exact test, Spearman’s rank correlation coefficient, and Kruskal–Wallis test were used for the statistical analysis. A *P*-value of less than 0.05 was considered to indicate a statistically significant difference between groups.

## Results

### Characteristics of the patients

**Figure 1.**
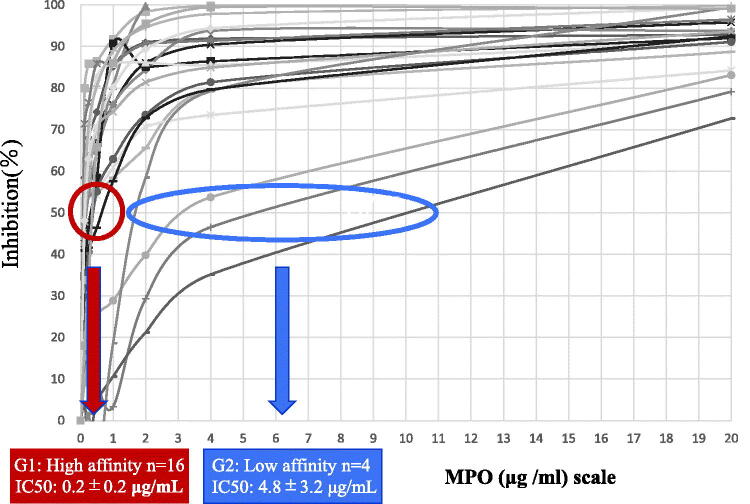
Affinity of myeloperoxidase (MPO)-anti-neutrophil cytoplasmic antibody (ANCA) for MPO evaluated by a competitive inhibition method using ELISA. According to the curves of the plot, the concentration of MPO causing 50% competitive inhibition (IC50) was calculated. The final results of MPO-ANCA affinity (IC50) of the 20 patients with MPO-ANCA-associated glomerulonephritis (AAGN) were classified into 2 groups, i.e., the high-affinity group (indicated by the red circle; 16 patients) and the low-affinity group (indicated by the blue oval; 4 patients).

The characteristics of all 20 patients (9 men and 11 women) and patients stratified by MPO-ANCA affinity are summarized in Table 1. The median eGFR was 17 mL/min/1.73 m^2^, and median proteinuria was 2.0 g/gCr．The degree of hematuria was semi-quantitatively scored into 4 grades: 0–4 RBC/high power field (HPF) = 1; 5–49 RBC/HPF = 2; 50–99 RBC/HPF = 3; and more than 100 RBC/HPF = 4.Patients in the MPO ANCA high-affinity group were significantly older, and showed significantly higher CRP levels than those in the low-affinity group. The median serum C3 level in total patients was 106 g/L, and a low serum C3 level was seen in only one patient (5%). The median serum C4 level in total patients was 28 g/L, and three patients (15%) showed low serum C4 levels. The profiles of MPO-ANCA affinity in all 20 patients is presented in Figure 1, and the results were classified into two types based on the pattern of each plot, as follows: the high-affinity group (G1) included 16 patients with an IC50 ≤ 1.0 μg/mL, and the low-affinity group (G2) included four patients showing an IC50 > 1.0 μg/mL. The median MPO-ANCA titer in G1 was 612 U/mL, whereas that in G2 was 132 U/mL. The difference in MPO-ANCA titer between the two groups was not statistically significant (*p* = 0.08).

**Table 1.** Baseline patient characteristics divided by MPO-ANCA affinity.

	Total *n* = 20	High affinity (G1) *n* = 16	Low affinity (G2) *n* = 4	P value
M/F (gender)	9/11	8/8	1/3	0.582
Age	70.5[67.0–74.5]	72.5[68.0–75.8]	65[41.3–67.8]	0.022
MPO-ANCA Affinity (μg/mL)	0.16[0.11–0.6]	0.14[0.10–0.34]	4.2[2.05–8.15]	<0.001
MPO-ANCA titer (EU)	426[130–900]	612[144–919]	132[76–295]	0.08
CIC-mRF (≧4.2 μg/mL)	13/20 (65%)	13/16 (81.3%)	0/4 (0%)	0.007
CRP (mg/dL)	3.3[1.2–8.7]	5.7[1.8–8.9]	0.4[0.1–1.5]	0.003
eGFR (mL/min/1.7 m^2^）	17[10–32]	20[10–32]	12[9.0–47]	0.385
C3 (mg/dL)	106[95–121]	111[94–126]	99[91–107]	0.411
C4 (mg/dL)	28[17–33]	27[17–32]	30[22–33]	0.357
Proteinuria (g/g Cr）	2.0[1.1–3.2]	2.5[1.3–4.1]	2.0[1.0–4.3]	0.892
Hematuria (score※)	3[1.0–3.0]	3[1.0–3.0]	2[1.3–2.8]	0.494

Abbreviations: MPO: myeloperoxidase; ANCA: anti-neutrophil cytoplasmic antibody; CIC-mRF: circulating immune complex assessed by monoclonal rheumatoid factor assay; CRP: C-reactive protein.

※ Hematuria scoring method.

1–4/HPF→0 5–49/HPF→1 50–99/HPF→2 100</HPF→4.

### Frequent detection of CICs in serum of patients with MPO-AAGN, especially in high-affinity group

As explained above, there were few AAGN patients with hypocomplementemia, but the results of CIC-mRF were found to be positive in 13 out of the 20 patients. Interestingly, all of the CIC-mRF-positive patients were in G1 (*p* = 0.007). On the other hand, only in three patients (15%) showed positive results on CIC-C1q (data not shown). All of these three patients also showed positive results on CIC-mRF, and therefore belonged to G1. Furthermore, there was a significant positive correlation between the data of the mRF assay and the C1q binding assay (R^2^=0.272, *p* = 0.002, data not shown).

### Increase in the serum levels of C5a and C5b-9, and their significant correlation with CICs in patients with MPO-AAGN

**Figure 2.**
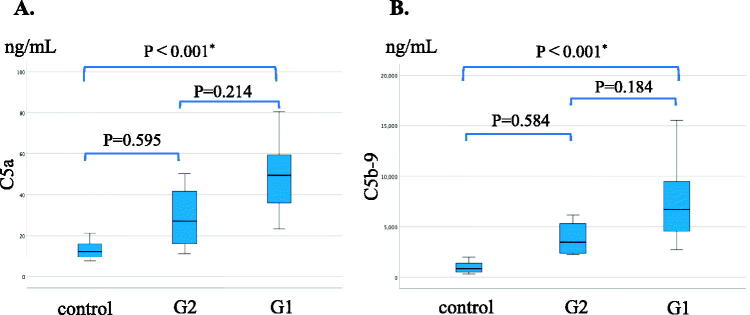
Serum levels of C5a and C5b-9 in MPO-AAGN patients and normal controls. A. Comparison of serum C5a levels in patients with MPO-AAGN in the low-affinity group (G1), high-affinity group (G2), and normal controls (control). B. Comparison of serum C5b-9 levels in patients with MPO-AAGN (G1 and G2) and controls. Statistical analysis was carried out by the Kruskal-Wallis test.

**Figure 3.**
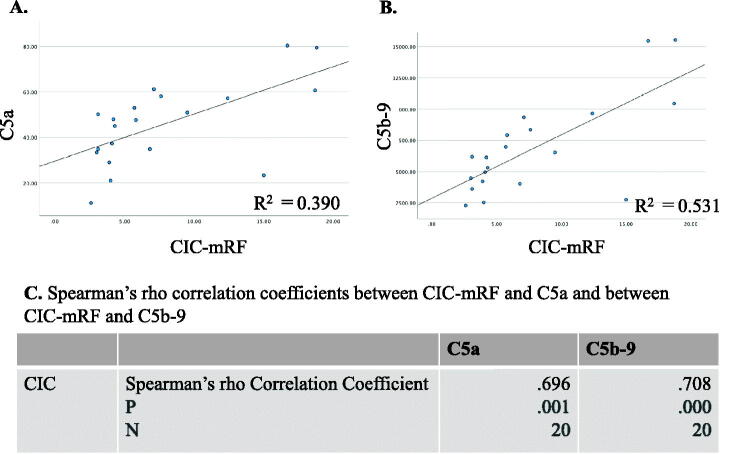
Correlation between levels of circulating immune complex (CIC) assessed by monoclonal rheumatoid factor assay (CIC-mRF) and serum C5a, and between levels of CIC-mRF and serum C5b-9. A. Scatter plot of CIC-mRF levels vs. C5a levels. B. Scatter plot of CIC-mRF levels vs. C5b-9 levels. C. Spearman’s rho correlation coefficients between CIC-mRF and C5a levels, and between CIC-mRF and C5b-9 levels, both of which showed a significant positive correlation.

**Figure 4.**
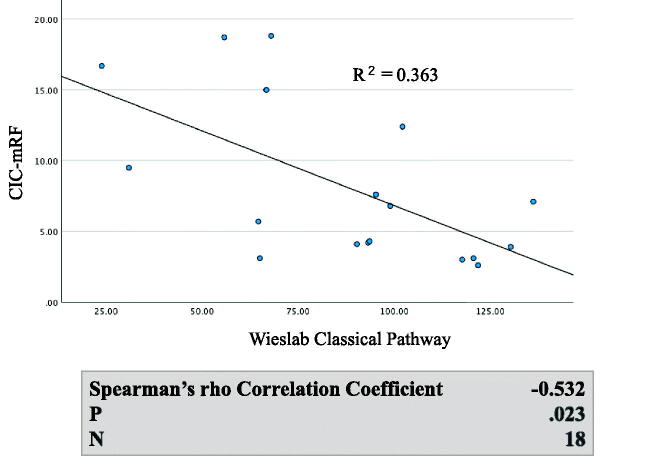
Correlation between levels of CIC-mRF and activation of the classical complement pathway measured by WIESLAB® Classical Pathway kit in serum. A. Scatterplot of CIC-mRF levels vs. activation levels of the classical complement pathway measured by WIESLAB® Complement System Classical Pathway. B. A significant negative correlation was found by the Spearman’s rho correlation coefficient.

Generally, both serum C5a levels and serum C5b-9 levels were significantly higher in MPO AAGN (G1 + G2) patients than in the healthy controls (C5a: 47.9[33.8–57.9] vs 12.3[9.4–16.7], *p* < 0.001; C5b-9: 6,162[4,071–9,102] vs 853[521–1,513], *p* < 0.001), and they also tended to be higher in the G1 group than in the G2 group, although the difference in both C5a and C5b-9 levels between G1 and G2 did not reach statistical significance (Figure 2). Moreover, levels of C5a and C5b-9 showed a significant positive correlation with CIC-mRF levels (*p* < 0.01) (Figure 3). On the other hand, a significant negative correlation was found between the levels of classical complement activation analyzed by the WIESLAB® Complement System Classical Pathway kit and the levels of CIC-mRF (*p* < 0.05, Figure 4).

### Light microscopic histological changes and serum complement related markers (CICs, C5a or C5b-9)

No significant correlation was found between the light microscopic histological changes (global sclerosis rate, crescent formation rate and IFTA score) with the levels of serum complement related markers (CICs, C5a or C5b-9) (data not shown).

### Frequent deposition of C4d, C5, and C5b-9 in glomeruli of patients with MPO-AAGN

All of the immunofluorescence staining results of the patients are listed in Table 2. Levels of IgG, IgA, IgM, C3c, and C1q, which were analyzed in routine examinations, were almost all negative, which is consistent with the diagnostic criteria of AAGN. Additional immunofluorescence staining performed on frozen sections included staining of C3 (pan specific: panC3), C5, C4d, C5b-9, factor Bb, MASP-1, MASP-2, and MBL. PanC3 was positive in 7 of the 20 patients (35%), and C5 was positive in 12 of the 20 patients (60%). Furthermore, C4d and C5b-9 were found to be positive in 75% and 85% of patients, respectively. In contrast, factor Bb was positive only in 20% of the patients, both MASP-1 and MASP-2 were positive only in 5% of the patients, and none of the patients were positive for MBL.

**Table 2.** Results of immunofluorescence staining in each patient.

CASE	IgG	IgA	IgM	C1q	C3c	panC3	C5	C4d	C5b-9	factor Bb	MASP-1	MASP-2	MBL
**1**	**−**	**−**	**−**	**−**	**−**	**−**	**±**	**+**	**+**	**+**	**−**	**−**	**−**
** 2**	**−**	**−**	**−**	**−**	**−**	**+**	**+**	**+**	**+**	**+**	**+**	**−**	**±**
** 3**	**−**	**−**	**−**	**−**	**−**	**+**	**++**	**++**	**++**	**±**	**−**	**−**	**−**
** 4**	**−**	**−**	**−**	**−**	**−**	**±**	**±**	**+**	**+**	**±**	**−**	**−**	**−**
** 5**	**−**	**−**	**−**	**−**	**±**	**+**	**+**	**±**	**+**	**±**	**−**	**−**	**−**
** 6**	**±**	**±**	**±**	**±**	**±**	**±**	**+++**	**++**	**++**	**−**	**−**	**−**	**−**
** 7**	**−**	**−**	**+**	**±**	**＋**	**±**	**+**	**+**	**+**	**−**	**−**	**−**	**±**
** 8**	**−**	**−**	**−**	**−**	**±**	**±**	**+**	**+**	**+**	**±**	**−**	**−**	**−**
** 9**	**−**	**−**	**−**	**+**	**−**	**+**	**±**	**−**	**+**	**+**	**±**	**−**	**−**
**10**	**+**	**+**	**+**	**−**	**−**	**±**	**±**	**++**	**+**	**−**	**−**	**−**	**±**
**11**	**±**	**−**	**−**	**±**	**±**	**±**	**++**	**+**	**+**	**−**	**±**	**±**	**−**
**12**	**−**	**−**	**+**	**−**	**−**	**±**	**±**	**+**	**±**	**−**	**−**	**−**	**−**
**13**	**−**	**−**	**±**	**−**	**−**	**±**	**±**	**±**	**±**	**±**	**±**	**＋**	**−**
**14**	**−**	**±**	**−**	**±**	**±**	**±**	**±**	**±**	**±**	**+**	**−**	**−**	**±**
**15**	**−**	**−**	**−**	**−**	**−**	**−**	**+**	**+**	**+**	**±**	**−**	**−**	**−**
**16**	**−**	**−**	**+**	**±**	**±**	**+**	**+**	**+**	**+**	**−**	**−**	**−**	**±**
**17**	**−**	**−**	**−**	**−**	**−**	**−**	**±**	**±**	**+**	**−**	**−**	**−**	**−**
**18**	**−**	**−**	**−**	**−**	**＋**	**++**	**++**	**+++**	**++**	**±**	**±**	**−**	**−**
**19**	**−**	**−**	**+**	**+**	**−**	**±**	**++**	**+++**	**+**	**−**	**−**	**−**	**−**
**20**	**−**	**−**	**−**	**−**	**±**	**+**	**+**	**+**	**++**	**−**	**−**	**−**	**−**
**positive rate (%)**	**5**	**5**	**25**	**10**	**10**	**35**	**65**	**75**	**85**	**20**	**5**	**5**	**0**

**Figure 5.**
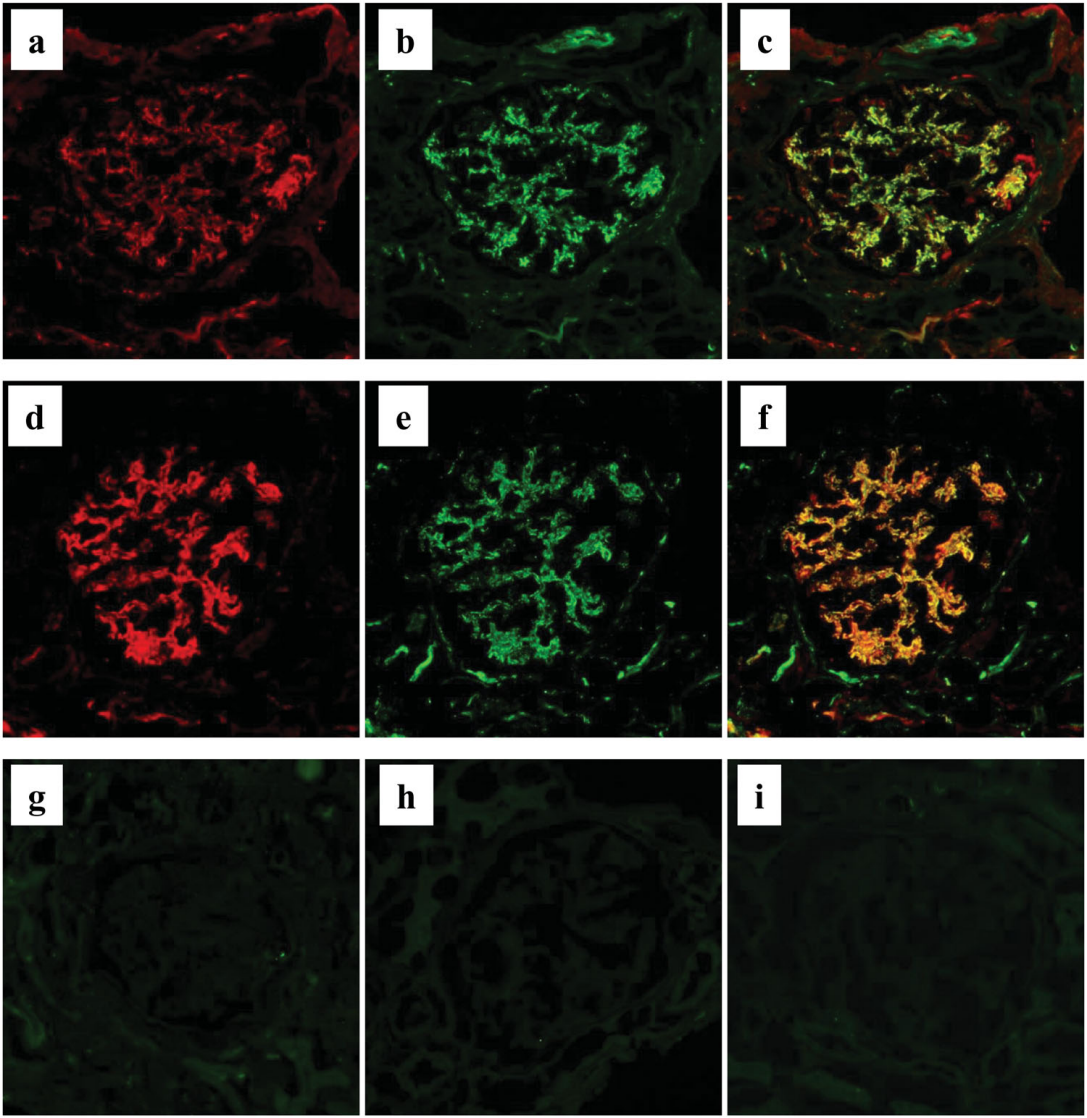
Immunofluorescence staining for complement components in MPO-AAGN. a–c. Representative image of direct double immunofluorescence staining for panC3 (a: Alexa Fluor 594: red) and C5 (b: Alexa Fluor 488: green) of a renal biopsy specimen of a patient with MPO-AAGN (case 3). A merged image is shown in (c). d–f. Representative image of indirect double immunofluorescence staining for C4d (d: Alexa Fluor 594: red) and C5b-9 (e: Alexa Fluor 488: green) on a renal biopsy specimen of a patient with MPO-AAGN (same patient as a–c). A merged image is shown in (f). Representative images of single IF staining for factor Bb neo antigen (g), mannan-binding lectin serine peptidase (MASP)-1 (h), and MASP-2 (i) on renal biopsy specimens of the same patient.

Double immunostaining demonstrated that regions of the glomerulus positive for panC3 and C5 were essentially similar; however, the staining intensity for C5 was much stronger than that for panC3 (Figure 5(a–c)). Furthermore, indirect double staining for C4d and C5b-9 also revealed that the positive regions in the glomeruli were similar (Figure 5(d–f)). Representative negative staining results of factor Bb, MASP-1, and MASP-2 in the same patient are shown in Figure 5(g–i).

## Discussion

In the present study, we found the existence of CIC with high frequency, and increased serum C5a and C5b-9 in patients with MPO-AAGN. Moreover, there was strong positive correlation between CIC levels and C5a and C5b-9 levels, and significant negative correlation between CIC levels and levels of the classical pathway kit. Histological analysis revealed the frequent presence of C4d, C5, and C5b-9 in glomeruli. These results suggest the involvement of immune-complex induced complement activation *via* classical pathway in the pathogenesis of MPO-AAGN.

Previous studies on mice demonstrated that ANCA does not cause RPGN in C5 or complement factor B knockout mice, but does so in C4 knockout mice [4]. Gou *et al.* reported that the renal deposition of factor Bb and urinary factor Bb levels are associated with the severity of renal injury [17]. Several studies have shown that low serum C3 levels, but not C4 levels, can estimate severe ANCA-associated vasculitis (AAV), and can predict poor renal outcome at diagnosis [5,18–22]. Furthermore, Sethi *et al.* evaluated the glomerular deposited protein by laser microdissection and mass spectrometry-based proteomic analysis, and found accumulation of complement factors, predominantly of the alternative pathway in MPO and PR3-ANCA positive as well as ANCA negative AAGN [7]. These studies suggest the important roles of the alternative complement pathway in the disease process of AAGN.

In contrast, there has also been a report showing that the urinary levels of C1q and MBL in patients with active AAV are significantly higher than that of normal controls, indicating that the classical or lectin pathways might also be activated in AAV [17]. Thus, it remains unclear as to which complement pathway is mainly involved in AAGN.

There are few reports that refer to CICs in AAGN, because AAGN is classified as pauci-immune GN based on the diagnostic criteria [3], and therefore, involvement of CICs has scarcely been reported. From this point of view, it is notable that more than half of the patients with AAGN showed the presence of CICs by the mRF assay in the present study. Although the frequency was lower, CICs were found to be positive also by the C1q binding assay in three patients (15%), all of whom were also positive for CICs by the mRF assay (data not shown). Furthermore, there was a significant positive correlation between the results of the C1q binding assay and those of the mRF assay (data not shown), suggesting the possibility that both assays detect similar molecules, although the sensitivity of the detection of the mRF assay is higher than that of the C1q binding assay. It is plausible that the use of the mRF assay may result in a higher CIC detection rate in AAGN patients.

As the presence of CICs analyzed by the mRF assay was significantly associated with a higher affinity of ANCA for MPO, we presumed that CICs were composed of MPO and MPO-ANCA. At present, however, we do not have definitive lines of evidence to support our idea. Therefore, the identification of the components of CICs as well as the mechanism of their formation in MPO-AAGN requires further study.

**Figure 6.**
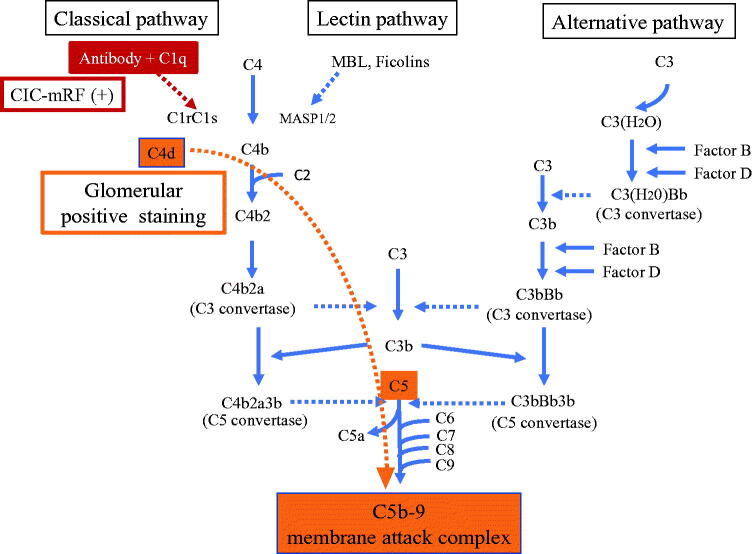
Outline illustration of complement activation system with our hypothesis of their role in MPO-AAGN. Serum and histological analysis suggest that CICs activate the classical complement pathway, leading to activation of the final common pathway and formation of C5b-9 in patients with MPO-AAGN.

The strong positive correlation between CIC levels assessed by mRF assay and serum C5a and C5b-9 levels, which are the end products of the complement pathway, suggest that CICs trigger the activation of the classical complement pathway, leading to activation of the final common pathway (Figure 6). Furthermore, a significant negative correlation was found between CIC-mRF levels and the results of the WIESLAB® Complement System Classical Pathway assay. As the results of this assay decreases in association with activation of the classical pathway, this significant negative correlation also supports the concept of CIC-induced classical pathway activation in MPO-AAGN. Furthermore, this notion was supported by the following pathological findings of the present study: frequent glomerular deposition of C4d, but minimal glomerular deposition of MASP-1, MASP-2, and MBL, which suggests that the classical complement pathway, and not the lectin pathway, plays an important role in the disease process of AAGN. In this context, however, the minor C1q staining was the conflicting result. We suspect that this may be due to the character of C4d molecule. Unlike other complement components such as C1q, C4d is known to bind covalently, and therefore firmly, to the tissue at the site of complement activation, and lingers stable in the site for long time [23,24]. Which means that C4d is more stable than C1q in affected tissue, therefore, the positive staining for C4d persists when that for C1q disappears after the complement activation had occurred at the site through classical pathway.

In the present study, we performed immunofluorescence staining for C3 using two different antibodies; one pan-specific for human C3, and the other specific for C3c (in routine evaluations). We found that the positive frequency was higher using the antibody against panC3 than using the antibody against C3c, suggesting the possible deposition of C3 components other than C3c, such as C3b and C3d. We also found strong and frequent staining using a fluorescently labeled antibody against the C5 molecule (eculizumab; Alexion Pharmaceuticals). This antibody is known to bind to C5 at a crucial portion for its activation, and blocks the disassociation of C5 into C5a and C5b [10], and is clinically used to treat a variety of diseases associated with complement activation, such as atypical hemolytic uremic syndrome, paroxysmal nocturnal hemoglobinuria, myasthenia gravis, and optic neuromyelitis. Positive staining with this antibody suggested the existence of a C5 molecule with a binding site for this antibody, i.e., the crucial portion for the disassociation-activation of C5 [25] in glomeruli, and therefore, may be direct evidence for the effectiveness of this antibody for this disease. Furthermore, a similar staining pattern in the glomerulus for panC3, C5, C4d, and C5b-9 also supports the concept that activation of the classical pathway leads to the activation of a final common pathway and the formation of effector molecule C5b-9 (membrane attach complex) in MPO-AAGN (Figure 6). To the best of our knowledge, this is the first study to analyze the association between CICs, AAGN, and MPO-ANCA affinity.

There remain unanswered questions as to why immune-complexes are not usually found by electron microscopy or immunofluorescence in renal tissues of patients with AAGN. One possible explanation would be the variation in the timing of hospitalization and renal biopsy in the disease course. However, Haas *et al.* analyzed 126 biopsies from AAGN patients, and found immune-complex deposits on electron microscopy in a little more than half of them [26]. Hilhorst *et al.* analyzed 187 renal biopsy samples from patients with AAGN, and detected the deposition of C3c, C3d, C4d, and C5b-9 in the majority of specimens [27]. Our outcomes are in line with this previous study, in which C4d and C5b-9 were found in more than 70% of the samples with minor positive staining for MBL or C1q. Regarding the reason as to why immune-complexes were not visible by electron microscopy, they hypothesized that local immune-complexes are quickly degraded in the early phase during the course of AAGN.

Our study has some limitations. The biggest one is the small number of patients analyzed, which reduced the power of the study and might produce some results inconclusive. Furthermore, the study was conducted in a single-center with inclusion of a single race, which made population and clinical practice biases inevitable.

In conclusion, our study indicates the important possibility that complement activation through the classical pathway occurs in the disease process of AAGN. Considering the rapid progress in complement controlling therapies, such as eculizumab [10,25] and avacopan [11], this finding may lead to the development of a new therapeutic strategy associated with restricted complement control in the near future.
